# Comparative analysis of haplotype association mapping algorithms

**DOI:** 10.1186/1471-2105-7-61

**Published:** 2006-02-09

**Authors:** Phillip McClurg, Mathew T Pletcher, Tim Wiltshire, Andrew I Su

**Affiliations:** 1 Genomics Institute of the Novartis Research Foundation, San Diego, USA; 2 Present address : The Scripps Research Institute, West Palm Beach, FL 33458 USA

## Abstract

**Background:**

Finding the genetic causes of quantitative traits is a complex and difficult task. Classical methods for mapping quantitative trail loci (QTL) in miceuse an F2 cross between two strains with substantially different phenotype and an interval mapping method to compute confidence intervals at each position in the genome. This process requires significant resources for breeding and genotyping, and the data generated are usually only applicable to one phenotype of interest. Recently, we reported the application of a haplotype association mapping method which utilizes dense genotyping data across a diverse panel of inbred mouse strains and a marker association algorithm that is independent of any specific phenotype. As the availability of genotyping data grows in size and density, analysis of these haplotype association mapping methods should be of increasing value to the statistical genetics community.

**Results:**

We describe a detailed comparative analysis of variations on our marker association method. In particular, we describe the use of inferred haplotypes from adjacent SNPs, parametric and nonparametric statistics, and control of multiple testing error. These results show that nonparametric methods are slightly better in the test cases we study, although the choice of test statistic may often be dependent on the specific phenotype and haplotype structure being studied. The use of multi-SNP windows to infer local haplotype structure is critical to the use of a diverse panel of inbred strains for QTL mapping. Finally, because the marginal effect of any single gene in a complex disease is often relatively small, these methods require the use of sensitive methods for controlling family-wise error. We also report our initial application of this method to phenotypes cataloged in the Mouse Phenome Database.

**Conclusion:**

The use of inbred strains of mice for QTL mapping has many advantages over traditional methods. However, there are also limitations in comparison to the traditional linkage analysis from F2 and RI lines. Application of these methods requires careful consideration of algorithmic choices based on both theoretical and practical factors. Our findings suggest general guidelines, though a complete evaluation of these methods can only be performed as more genetic data in complex diseases becomes available.

## Background

The discovery of genes that directly affect human health is an active area of biomedical research. Although studies have been historically biased toward the role of individual genes in disease phenotypes, most complex diseases are caused by the influence of numerous genes with lesser individual effects. Current efforts in mapping quantitative trait loci (QTL) seek to unravel these complex mechanisms through the identification of one or more genetic loci that influence specific phenotypes.

All QTL mapping approaches have three components in common: a population of individuals with a measurable phenotypic diversity, a measure of the genotypic diversity present in that population, and a statistical method to assess the association between the phenotype and genotype. Over recent decades, much focus has been directed toward "classical" QTL mapping techniques in the mouse, which use phenotypic and genotypic diversity generated using F2 intercrosses or backcrosses and an interval mapping method introduced by Lander and Botstein [1]. This approach has been successfully used to map thousands of QTL in rodents for a wide range of phenotypes, ranging from taste preference to disease susceptibility. However, because this approach uses mouse crosses to generate phenotypic and genotypic diversity, genetic replicates of the F2 population cannot be easily produced. Therefore, genotyping of each F2 animal is necessary after the initial breeding step, which makes traditional QTL mapping both expensive and time-consuming, often requiring months or years to complete. Furthermore, of the thousands of QTL that have been identified, only a small percentage have been characterized at the molecular level, in part because of the large size of QTL intervals [2].

Recombinant inbred (RI) panels of mice [3] in which the genomes of a pseudo-F2 population are fixed have also been used for QTL mapping. These strains have the advantage that isogenic progeny can be easily maintained in the laboratory, and consequently, genotyping in individual strains can be applied to many phenotypes. Also, RI strains contain more recombination events relative to F2 animals, potentially improving QTL resolution. However, since RI panels are also expensive and time-consuming to generate, the availability of specific crosses is currently limited.

Here, we describe a class of QTL mapping methods which uses the existing phenotypic and genotypic variation that occurs in common laboratory inbred mouse strains for association studies (previously referred to as "*in silico *mapping" [4]). Over the past century of breeding and inbreeding to produce the commonly used modern laboratory strains of mice, wide variations in phenotypic traits have been observed. Efforts to catalog these inter-strain phenotypic differences are well underway (e.g., MPD; [5,6]). The genotypic structure of these strains is also being elucidated through efforts at dense mapping of single nucleotide polymorphisms (SNP), and variance among these strains is emerging in the form of haplotype structure [7-10]. It has been hypothesized that these inbred mouse strains have the necessary experimental requirements to facilitate QTL mapping [4,9]. This hypothesis suggests that phenotype-specific mouse crosses would not be required for the initial identification of QTL, and that large-scale genotyping efforts could be generated and combined in a phenotype-independent manner. Furthermore, this method would be applicable to organisms in which controlled breeding is not feasible.

Other association mapping efforts using a diverse panel of inbred strains have been reported [4,11,12]. Recently, we reported results from QTL mapping in inbred strains of mice based on a haplotype association strategy [9]. Briefly, 10,990 SNPs spaced at ~300 Kb intervals were identified from the Celera Mouse SNP database. The genomic DNA of 48 strains of mice (including the 40 priority strains of the Mouse Phenome Project) was genotyped across this SNP set, producing a total of 470,407 allele calls. These data were used to map known and novel QTL for several monogenic traits, as well as complex traits such as sweet taste preference, high-density lipoprotein cholesterol (HDLC) levels, and gallstone formation. Independent investigators have also used our haplotype association method to refine a QTL region from an F2 intercross study [13].

In this manuscript, we describe in detail a comparison of various algorithmic approaches to haplotype association mapping, in terms of both statistical rigor and success in reproducing known biology. In particular, we address issues of haplotype size and structure, parametric and non-parametric estimation of significance, and control of multiple testing error. Finally, we conclude with a description of initial efforts to apply our haplotype association mapping algorithm to the numerous phenotypes stored in the Mouse Phenome Database.

## Methods and results

### Test data sets

For the purposes of evaluating our haplotype association mapping algorithms, we considered two phenotypes for which the genetic determinants are relatively well-characterized: sweet taste preference and HDLC. Sweet taste preference is a relatively simple quantitative trait for which several QTL have been identified [14]. Furthermore, one QTL region has been narrowed to a specific quantitative trait gene (QTG), called *Tas1r3*, which is responsible for 30% of the variation observed in the sweet taste preference phenotype [15]. HDLC is a complex quantitative trait for which many QTL have been identified using traditional cross-based QTL mapping [16]. Forty-two percent of the mouse genome falls within a known QTL confidence interval. Since in most cases these QTL have not been refined to corresponding QTG, and since the cross-based QTL mapping has its own false positive and false negative rates, this system is not the ideal "gold standard" for the assessment of specificity and sensitivity. Nevertheless, as one of the most well-studied multigenic and quantitative traits, HDLC levels may be the best available benchmark for evaluating haplotype association mapping results in a complex phenotype.

### Calculating association scores

#### 1. Single-marker mapping (SMM)

**Figure 1:**
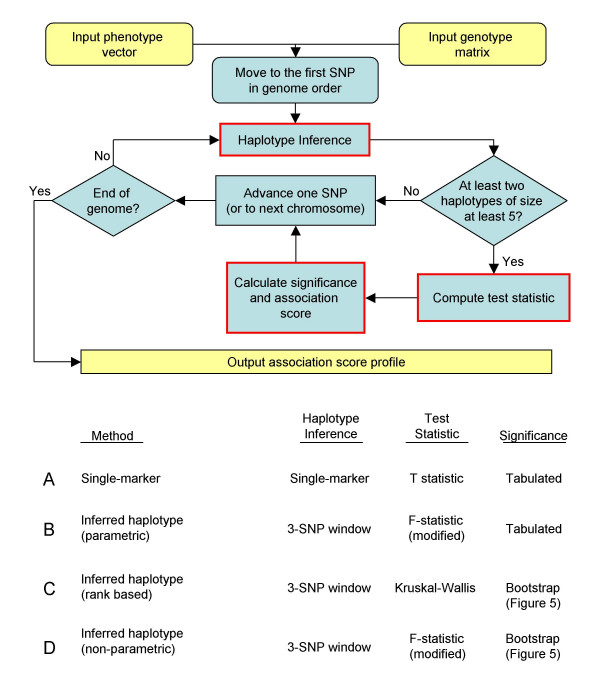
**Algorithmic framework for calculation of association scores. **This flowchart outlines the basic algorithm common to the association methods examined. All variations use as input a vector of phenotype values across multiple mouse strains (e.g., HDLC levels, sweet taste preference) and a genotype matrix of allele calls across our 11 K SNP set. At each position in the genome, these algorithms use the local genotype data to generate inferred haplotypes. A test statistic is computed and its significance is estimated to detect groups with different mean phenotypes. This framework is used to evaluate our (A) single-marker method (SMM), (B) parametric inferred haplotype method (IH-P), (C) Kruskal-Wallis based inferred haplotype method (IH-KW), and (D) bootstrap inferred haplotype method (IH-B).

**Figure 2:**
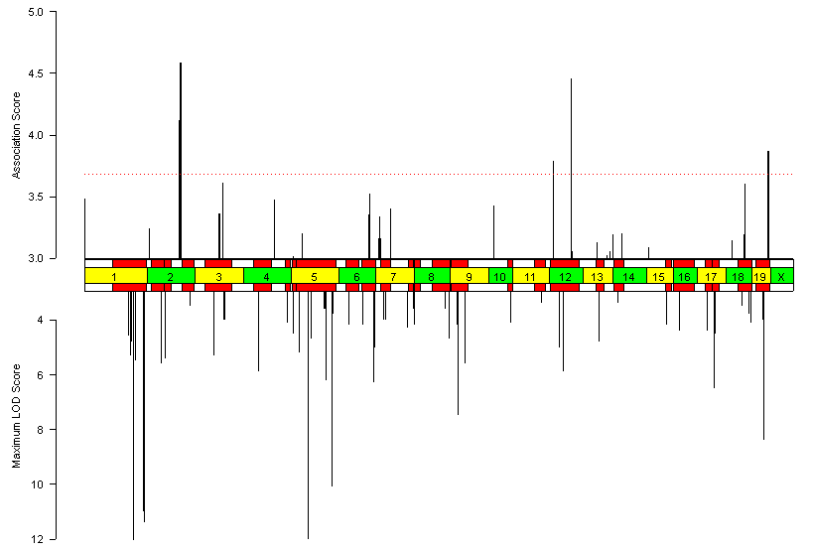
**Results of the single-marker mapping (SMM) method for HDLC. **The upper bar chart shows the computed HDLC phenotype association profile using the parametric SMM method. The lower bar chart shows the maximum LOD scores at previously known intervals (95% confidence intervals shown as red rectangles) [16]. The x-axis indicates the genomic axis, where chromosomal boundaries are indicated by the center bar. The maximum LOD scores are cut off at 12. Association scores below 3 and LOD scores below 3.3 are not shown. Peaks on the X chromosome are ignored, and multiple peaks within a 5MB window are only counted once. Of the twenty loci with the highest association score, eleven intersect previously known QTL intervals.

The simplest method of computing associations between genotype and phenotype is single marker mapping (SMM), in which each SNP position is considered separately (Figure 1A) [17]. Since each SNP is biallelic across inbred strains, a t-test is used to measure the strength of association between genotype and phenotype. For clarity, all p-values are transformed using -log_10_(p-value), henceforth referred to as the "association score". Since the segregation of strains into genotypic groups varies widely over loci, the association score and not the locus-specific test statistic is compared between loci. The SMM can successfully map the sweet taste preference loci [see Additional file 1]. For the HDLC phenotype, of the top twenty peaks identified by SMM, eleven intersect a previously known QTL interval and nine do not (Figure 2) (p < 0.171, estimated using binomial distribution).

#### 2. Mapping by inferred haplotype structure, parametric model (IH-P)

**Figure 3:**
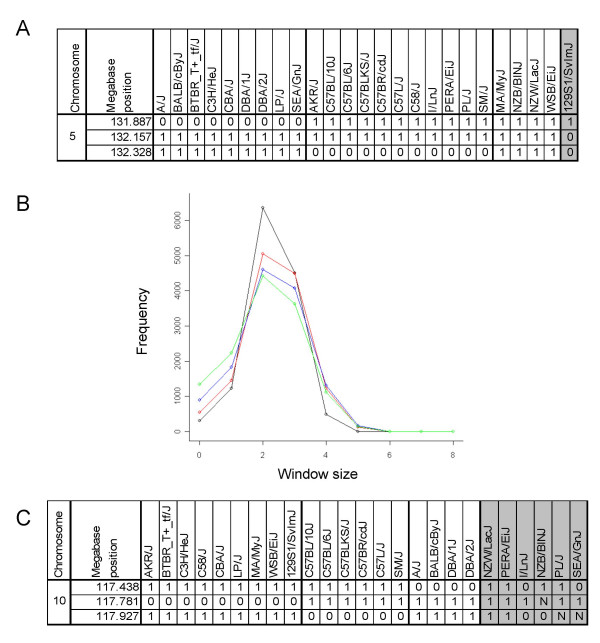
**Local haplotype inference. **(A) A three-SNP window is shown across 25 strains. A "0" denotes a minor allele (at the bi-allelic locus) and "1" the major allele. An "N" denotes no information for that strain, and strains lacking genotype data at any SNP position in a given window are not included in association computations at that locus. The three-SNP window shown contains three inferred haplotype groups with sizes of nine, twelve, and four strains per group. Groups with fewer than three strains are not considered in the analysis. This three-SNP window, which overlaps a previously known QTL, illustrates why the genotype data at a single locus is insufficient to model the genomic structure across inbred strains. (B) A histogram showing the number of haplotype groups for window sizes of two SNPs (black), three SNPs (red) four SNPs (blue) and five SNPs (green) is shown. Inferred haplotype groups with less than three members are excluded from the calculations and are not counted here. While two-SNP windows often show the maximum of four haplotype groups, larger SNP window sizes rarely exceed eight haplotype groups. This observation suggests that three-SNP windows are most appropriate for this strain set and SNP data. (C) A three-SNP window is shown with highly unequal group sizes and unequal variances. This locus is identified as significant by IH-KW but not IH-P.

Although the SMM strategy can be successful at identifying previously known QTL, the biallelic structure of inbred strains at a single SNP locus allows only two genetic groups to be modeled. The assumption of two parental haplotypes is well-suited to the context of QTL mapping based on F2 or RI populations. However, when using the panel of inbred strains of mice, inspection of allele patterns across multiple loci suggests that the genetic structure in many cases is more complex (Figure 3) [8-10]. For example, Figure 3A shows a region on mouse chromosome 5 contained in a previously described QTL interval for HDLC [16]. Over this three-SNP window, three predominant genotype patterns are observed. The genotype at one biallelic SNP is insufficient to model this genomic structure, explaining why this locus was not detected using the SMM strategy.

These observations led us to define an inferred haplotype group as a set of strains with an identical genotype pattern over a local window of SNPs. A genome-wide analysis of inferred haplotype groupings shows that the number of inferred haplotype groups can frequently exceed even four (the number able to be encoded by a window of two biallelic SNPs) but rarely exceeds eight (encoded by a three-SNP window) (Figure 3B). Therefore, we chose to define inferred haplotype groups based on three contiguous SNP loci (the use of four-SNP windows was found empirically to recover fewer known QTL, data not shown). Two strains are defined to be in the same inferred haplotype group if and only if their genetic pattern across three adjacent SNPs is identical. Based on our current SNP density, this three-SNP window reflects an average inferred haplotype size of 900 kb. Our previous studies suggest that haplotype block sizes can commonly be this size or larger [8], although others have reported more fragmented structures in studies in specific genomic regions [7,10]. Nevertheless, efforts to increase the density of SNP data will improve the ability of our method to interrogate regions of small haplotype size.

Using these rules, strains are grouped according to inferred haplotype at each three-SNP window. To reduce the occurrence of spurious associations, we require that two inferred haplotype groups of at least five strains each exist at a given locus to be considered. Groups consisting of less than three strains are not considered in the analysis, and strains with missing data are removed from the analysis at this locus. Based on these groupings of inferred haplotype, the F-statistic from analysis of variance (ANOVA) tests the significance of the genotype/phenotype association at a given locus.

**Figure 4:**
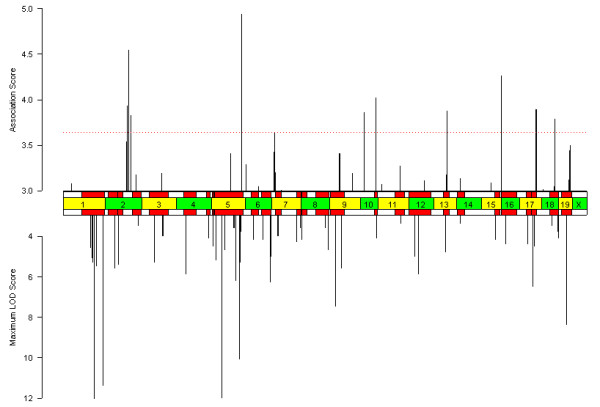
**Results of the inferred haplotype parametric (IH-P) association method for HDLC. **As in Figure 2, the top bar chart shows the association score profile, and the bottom bar chart shows the peak LOD scores and significant QTL intervals described previously for HDLC. Of the twenty loci with the highest association score, thirteen intersect previously known QTL intervals.

Given this model of genotypic structure, we first chose to estimate p-values using an inferred haplotype parametric (IH-P) approach based on a parametric model of ANOVA (Figure 1B). Here our test statistic is a modified F-statistic, which differs from the standard F-statistic by a weighting factor that accounts for the population structure in the panel of inbred strains (as described in [9]). The results of the HDLC phenotype in this context are shown in Figure 4 (results for sweet taste preference; [see Additional file 2]). The locus for sweet taste preference is correctly mapped, and of the top twenty peaks identified by IH-P for HDLC, thirteen intersect a previously known QTL interval and seven do not (p < 0.033).

#### 3. Mapping by inferred haplotype structure, Kruskal-Wallis model (IH-KW)

**Figure 5:**
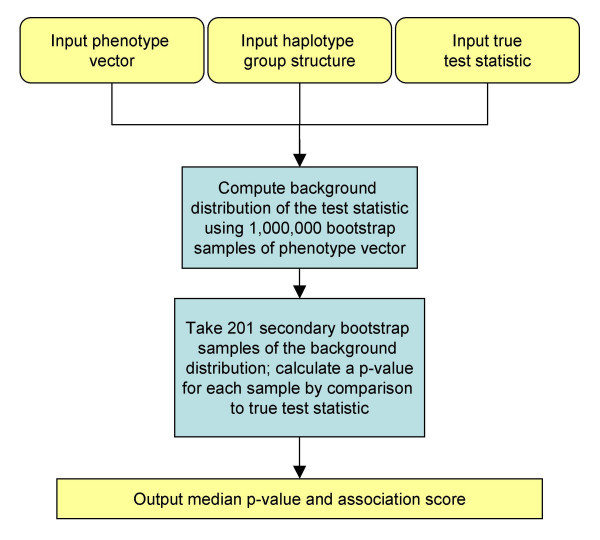
**Bootstrap estimation of p-values and association scores. **Rather than inferring the significance of a test statistic using tabulated values that assume the structure of the background distribution, this method empirically computes a background distribution based on bootstrapped phenotype vectors. Furthermore, confidence intervals are estimated using the double bootstrap procedure described.

Figure 3C shows a three-SNP window on chromosome 10 with a known association with the phenotype [16], but this locus is not identified as significant by either the SMM or IH-P method. This window contains inferred haplotype groups with unbalanced sizes and variances that are significantly different, violating the parametric assumptions in the IH-P method. To reduce the dependence on these assumptions, we used a rank based test statistic that uses the inferred haplotype structure. The inferred haplotype Kruskal-Wallis (IH-KW) algorithm is depicted in Figure 1C. The Kruskal-Wallis test statistic is computed at each locus, and the significance is calculated using a bootstrap distribution as described in Figure 5. By using 1,000,000 bootstrap samples, we model the background distribution of the test statistic at a given locus and use this sample to assess the p-value of test statistic calculated from the true phenotype values. To reduce the variance in the p-values, we also employ 201 double bootstrap samples, as described below and in Figure 5.

**Figure 6:**
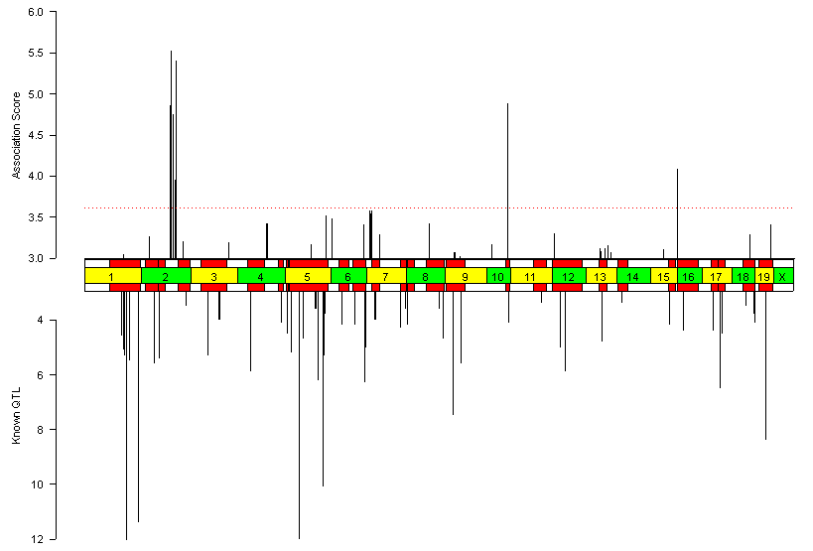
**Results of the inferred haplotype Kruskal-Wallis (IH-KW) association method for HDLC. **As in Figure 2, the top bar chart shows the association score profile, and the bottom bar chart shows the peak LOD scores and significant QTL intervals described previously for HDLC. Of the top twenty peaks, twelve intersect a previously known QTL interval.

The results of association mapping using the IH-KW method are shown in Figure 6 (results for sweet taste preference; [see Additional file 3]). The locus controlling sweet taste preference is again correctly identified, and of the top twenty peaks identified by IH-KW for HDLC, twelve intersect a previously known QTL interval and eight do not (p < 0.081).

#### 4. Mapping by inferred haplotype structure, bootstrap model (IH-B)

**Figure 7:**
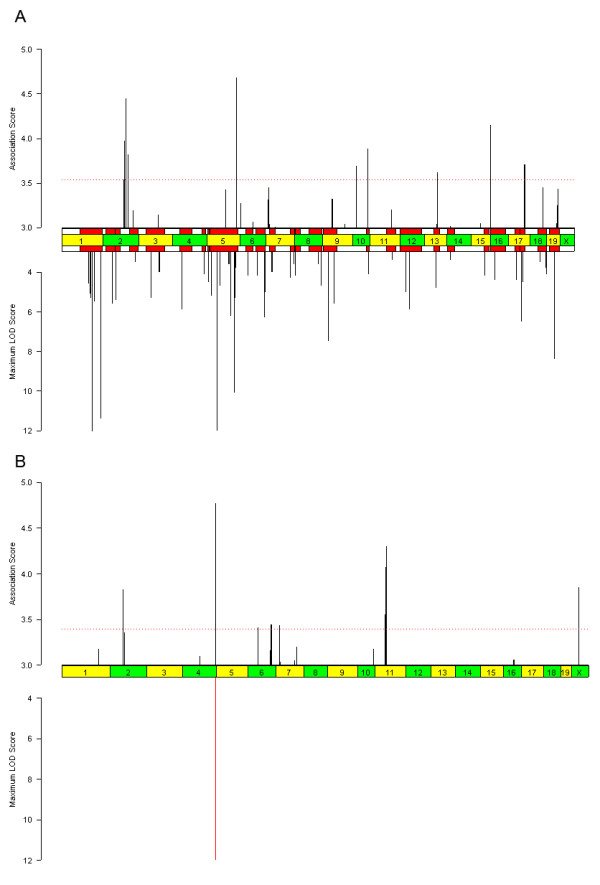
**Results of inferred haplotype bootstrap (IH-B) association method for HDLC and sweet taste preference phenotypes. **As in Figure 2, the top bar chart shows the association score profile, and the bottom bar chart shows the peak LOD scores and significant QTL intervals described previously for (A) HDLC and (B) sweet taste preference. In addition, the horizontal red dotted line is a gFWER threshold described in the section on "Controlling for multiple testing". Of the top twenty peaks for HDLC, fourteen intersect a previously known QTL interval and six do not.

Since rank-based methods discard the quantitative information present in the data, we next examined the use of a nonparametric inferred haplotype bootstrap (IH-B) method to calculate association scores (Figure 1D). In contrast to the Kruskal-Wallis metric, bootstrap methods of estimating significance utilize the original structure of the data. At each three-SNP window, we compute the modified F-statistic used in the IH-P approach and use the bootstrap protocol described in Figure 5 to calculate the significance. The results of IH-B applied to the HDLC and sweet taste preference phenotypes are shown in Figure 7. Of the top twenty peaks identified by IH-B for HDLC, fourteen intersect a previously known QTL interval and six do not (p < 0.011).

### Controlling for multiple testing

#### 1. Statistical power and false discovery rates

It is important to note that association scores only indicate candidate genomic regions that may contain a gene that influences a given phenotype. The causal relationship between a specific gene and a phenotype can only be confirmed using experimental techniques (e.g., mouse knockouts, complementation experiments). A method of assessing power, the false discovery rate, and/or the false positive rate of our method would therefore be of practical value for evaluating this haplotype association mapping algorithm.

Accurate estimates of power for our haplotype association method have not yet been determined since the distributions that arise from our methodology in many cases do not satisfy parametric assumptions of existing methodology in the literature. Methods proposed to estimate the FDR [18,19] have used the distribution of p-values over all hypothesis tests to estimate significance cutoffs. However, in our genome-wide association scans, approximately 11,000 hypothesis tests are conducted at overlapping windows of SNPs. Because neighboring windows share common alleles, each hypothesis test is highly non-independent, resulting in overly conservative FDR thresholds using the methods cited above.

#### 2. Family-wise error rate (FWER)

While the assessment of false positive rate is less desirable than estimates of power or FDR, it is the only assessment for which a mathematically justifiable approach exists for its computation. The method we utilize applies to multiple testing situations yet makes no assumptions about the independence of the multiple hypothesis tests.

**Figure 8:**
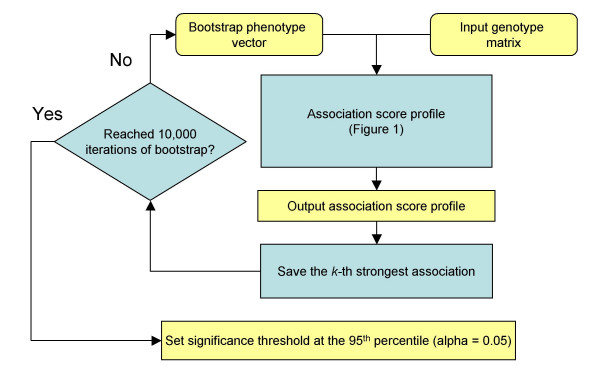
**Flowchart describing FWER *(k *= 1) and gFWER *(k *> 1) algorithms. **Genome-wide bootstrap iterations are used to control for multiple testing error. Bootstrap samples of the phenotype vector are used to simulate the random background distribution for association score profiles. For each bootstrap sample, an association method shown in Figure 1 is used to compute one observation of the background distribution. For computational efficiency, gFWER calculations only utilize 500,000 primary and 101 secondary bootstrap samples when estimating significance (Figure 6). Additionally, instead of reporting the median (Figure 6) the maximum is reported to satisfy the hypothesis of the null dominance criterion [23]. When k = 1, this flowchart describes the FWER algorithm. For k > 1, this procedure describes the gFWER approach outlined by Dudoit et al.

We implemented an algorithm to compute a family-wise error rate (FWER) of our association scores [20,21] (Figure 8, *k *= 1). Given a set of hypothesis tests, the FWER threshold is defined by the probability of observing even one false positive (Type 1 error) occurring among any of the hypothesis tests (at a significance level α, typically α = 0.05). The bootstrap process outlined in Figure 8 is repeated 10,000 times with a bootstrapped phenotype vector, and each iteration produces a simulated association score profile. The bootstrap simulation results in a background distribution of association score maxima, and the critical value for the rejection region is defined as the α = 0.05 percentile of this distribution. In all cases our bootstrap is uniform sampling with replacement.

It has been understood for some time that the FWER is an extremely strict method of defining the critical value for the rejection region [22]. Using any of our association methods, we find that the FWER threshold produces no significant associations for HDLC, despite the biological observation that many of the top loci overlap with previously known QTL. In the process of protecting against false positives (ensuring absolute specificity), FWER sacrifices sensitivity and effectively eliminates the power of these association methods.

#### 3. Generalized FWER (gFWER)

To generate significance thresholds that report practically useful associations, the rejection region must be relaxed by increasing the tolerance of false positives. In the context of multiple testing, the generalized family wise error rate (gFWER) is the probability of at least *k+1 *Type I errors occurring among any of the hypothesis tests [23]. Our implementation of the method proposed by Dudoit et al. is also illustrated in Figure 8, where *k *> 1. In contrast to the FWER approach which records the genome-wide maximum, we record the *k*th largest association score genome-wide, where *k *is the number of tolerated Type I errors. To insure the null dominance criterion of gFWER holds [23], we estimate the supremum (least upper bound) of the random association score generated at each locus by using the maximum association score generated from secondary bootstraps samples (instead of the median denoted in Figure 5). In this way, we create a reference distribution that conservatively estimates the distribution of the *k*th largest values. In the end we have a reference distribution which can be adjusted to tolerate any level of false positives, but allows for asymptotic control of the gFWER.

**Figure 9:**
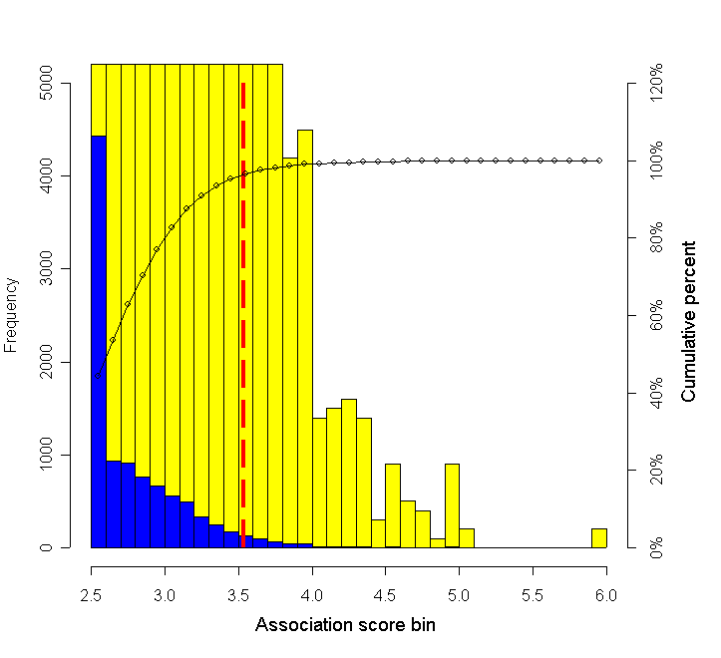
**gFWER reference distribution (*k *= 10) for HDLC. **The genome-wide bootstrap approach outlined in Figure 8 is used to generate a distribution of background association score profiles. Here, we show the background distribution for *k *= 10. For computational efficiency, at each combination of SNP window and bootstrap sample, association scores less than 2.5 are set at an upper bound of 2.5 (reducing the number of bootstrap iterations required in Figure 6). The black line shows the cumulative distribution of association scores. The vertical red line is the αg 0.05 percentile of these 10,000 values and is set at the gFWER threshold (*k *= 10). For visualization, the blue bars show the raw frequency values, and the yellow bars show a 100 × magnification.

For HDLC, the bootstrapped reference distribution of *k *= 10 for IH-B is shown in Figure 9. The critical value for the rejection region is set at the α = 0.05 percentile. The gFWER thresholds for both the HDLC and sweet preference phenotypes are shown in Figure 7 (red dotted line). Importantly, by allowing some tolerance of false positives in the control of multiple testing, several significant loci (more than the number of tolerated false positives) are identified that overlap with previously described HDLC QTL. Table 1 shows the significance thresholds using IH-B at various tolerances for false positives (α = 0.05) and the number of loci that exceed the threshold. As noted previously, the FWER threshold (*k *= 1) reveals no significant associations. It is important to note from Table 1 that increasing values of *k *lead to alternate methods of estimating FDR. For example, at false positive tolerances of *k *= 5 or less, the number of loci declared "significant" is consistent with a random phenotype (FDR ≈ 100%). However, at *k *= 10 and *k *= 20, the number of significant loci exceeds the tolerance for false positives, indicating that there exists loci with association scores that are significantly higher than random. While the false discovery rates may seem relatively high, they are consistent with what one expects for a complex trait in which the marginal genetic contribution of any single gene can be quite low.

**Table 1:** Loci exceeding gFWER thresholds for inferred haplotype bootstrap (IH-B) for HDLC phenotype. gFWER thresholds for association scores for *k = *1,3,5,10,20 and 30 and the number of loci exceeding these thresholds for the HDLC phenotype using IH-B.

*k*-gFWER	Threshold (alpha = .05)	Loci Above Threshold	FDR
1	4.92	0	>100%
3	4.19	2	>100%
5	3.91	4	>100%
10	3.53	17	52.9%
20	3.19	34	55.9%
30	3.00	46	63.0%

### Mining the mouse phenome database

We have also applied our algorithms to a sampling of phenotypes in the Mouse Phenome Database (MPD; [5,6]}). Currently there are nearly 1000 phenotypes stored in the MPD across a wide range of phenotypic categories. We chose a subset of seventeen phenotypes on which we ran all of our haplotype association methods. The phenotype data for males and females were analyzed separately. Genome-wide significance was computed with the gFWER method, *k *= 10. Table 2 shows the number of significantly associating loci for each phenotype and method.

**Table 2:** Association mapping to phenotypes in the Mouse Phenome Database. The number of loci that exceed the gFWER threshold (*k *= 10) is shown for each method and selected phenotypes from the Mouse Phenome Database.

Phenotype			Number of loci above gFWER k = 10			
MPD ID	Name	Sex	SMM	IH_P3	IH_KW	IH_B
mpd103	BMD	f	1	5	10	8
mpd103	BMD	m	1	3	4	2
mpd103	CaCl2 pref7	f	15	16	20	18
mpd103	CaCl2 pref7	m	19	13	43	15
mpd103	fat wt	f	5	11	7	16
mpd103	fat wt	m	0	1	0	0
mpd103	NaCl pref25	f	6	3	3	5
mpd103	NaCl pref25	m	4	4	3	7
mpd143	INS 18	f	1	1	8	2
mpd143	INS 18	m	4	3	4	3
mpd143	LEP 18	f	0	0	2	0
mpd143	LEP 18	m	0	0	0	2
mpd29	estHCC	f	4	3	0	3
mpd29	estHCC	m	5	0	1	1
mpd29	freeHCC	f	11	15	15	16
mpd29	freeHCC	m	1	1	2	2
mpd29	totHCC	f	17	13	11	19
mpd29	totHCC	m	2	0	1	0
mpd62	cHGB	f	0	0	0	0
mpd62	cHGB	m	20	9	14	9
mpd62	LYM	f	20	25	175	25
mpd62	LYM	m	3	10	49	15
mpd62	MONO	f	5	1	0	2
mpd62	MONO	m	7	15	28	14
mpd62	NEUT	f	471	617	213	637
mpd62	NEUT	m	299	185	59	250
mpd62	WBC	f	2	2	1	2
mpd62	WBC	m	3	6	14	3
mpd91	ASR 80	f	11	6	3	7
mpd91	ASR 80	m	6	2	2	4
mpd91	PPI 20	f	8	3	1	3
mpd91	PPI 20	m	1	1	1	1
mpd99	TG	f	15	5	1	6
mpd99	TG	m	40	17	53	22

Since these phenotypes are even less well-characterized than the examples we used in the method development (HDLC and taste preference), detailed analyses of specificity and sensitivity are not possible. Nevertheless, some general trends are apparent. First, more than ten significant loci (our tolerance threshold for false positives) by any method were observed in less than half (15 out of 34) of the phenotypes examined. Several factors could explain the lack of associations in the majority of phenotypes. There could be a gene or genes that truly affect the phenotype, but the genotyping data in this region has missing data or is not dense enough to accurately represent the haplotype block structure. Alternatively, the marginal affect of any one gene on the overall phenotype could be too small to detect using these methods. It is also observed that regions of greater haplotype diversity are not interrogated well by these methods due to the loss of power resulting from decreased populations in each haplotype group.

Second, in cases where more than ten loci are observed, the IH-KW method is usually the method which reports the largest number of associations. These associations are likely due to the population structure effects described above. Although the modified F-statistic used in the IH-P and IH-B methods accounts for haplotype groups that are dominated by closely related strains, the Kruskal-Wallis statistic is not easily modified in a similar way. Therefore, it is likely that the high number of associations is a result of association to the background genetic structure among the inbred lines. Two other observations corroborate this hypothesis. The use of an unweighted F-statistic also increases the number of observed associations, and the discrepancy is most notable in phenotypes (e.g., LYM) in which the group of strains with a highest degree of genetic similarity (typically related to the C57 lineage) contains clustered and extreme values (data not shown). In the most extreme example, NEUT, all methods report unusually long stretches of associating loci, indicating that even the modified F-statistic is not sufficient to counteract the strong correlation between phenotype and population structure.

## Discussion

QTL mapping in rodents has been an important strategy for narrowing the expansive genome to relatively small regions of the genome containing genes relevant to a phenotype of interest. To date, the majority of QTL have been identified using populations based on F2 crosses. However, these methods are time-consuming and expensive. Furthermore, only a small percentage of the QTL identified using F2 crosses have been mapped down to the causative gene or polymorphism, at least in part due to the comparatively large size of the QTL regions.

Here, we presented a comparative analysis of methods that utilize the genetic and phenotypic diversity present in common laboratory inbred mouse strains. The potential advantages of this type of approach are two-fold. First, phenotype-specific mouse crosses are not required to generate the required genetic and phenotypic diversity for initial QTL identification. Phenotype data still need to be measured on the panel of inbred mouse lines, but assuming an appropriate range of phenotype values exists, the rest of the association analysis can be performed *in silico*. Second, large-scale genotyping efforts can be generated and combined in a phenotype-independent manner, making this approach amenable to collaborative efforts that will benefit the entire mouse genetics community. Furthermore, given the existing data sets that we and others have produced, our haplotype association mapping method allows association studies to be quickly performed over a number of available phenotypes (MPD, for example).

QTL mapping has also been performed using RI lines, which also have the benefit of combining genotype data in community efforts. However, because commonly available RI lines are derived from only two parents, regions in which the parental strains are identical-by-descent (IBD) cannot be probed for QTL. For example, when comparing C57/BL6J and DBA (parents in the BXD RI panel), only 6292 loci have a different inferred haplotype. In contrast, the full panel of laboratory inbred mouse strains interrogates 11182 loci, even after filtering out loci with trivially small haplotype group sizes. In addition, QTL mapping by RI lines is also currently constrained by the limited availability of specific crosses.

Although the set of inbred mouse strains used in our analysis contains greater genotypic and phenotypic diversity compared to the currently available RI lines, the proposal by the Complex Trait Consortium to create additional 1000 RI strains could serve as an even more powerful resource for genome-wide association algorithms [3]. Since these strains will be derived from crosses of eight parental strains, they will certainly represent an equally broad genotypic and phenotypic diversity as our panel of inbred strains. In addition, the controlled randomization of the genome will lead to a more controlled population structure than is currently found in the common laboratory inbred strains.

Here, we have explored variants of the association mapping algorithm we originally reported [9] using different test statistics and methods of calculating significance in currently available inbred strains. In addition, we have investigated the use of generalized FWER thresholds for setting genome-wide significance thresholds. Although the lack of a true gold standard prevents a definitive comparison between these methods, two general trends are observed that can likely be extrapolated to all association mapping in inbred strains. First, since the haplotype block structure in inbred strains is complex relative to RI or F2 populations, the use of multi-SNP windows to assign haplotype groups is more appropriate than simply using the genotype at a single locus. Second, because population structure is clearly evident in these inbred lines, methods to account for that structure must be incorporated into association mapping algorithms. Here, we utilize a modified F-statistic that factors into the calculation the average pairwise genetic similarity within a haplotype group.

Despite the potential advantages of haplotype association mapping, the limitations in the experimental design relative to traditional cross-based QTL mapping must be noted. As noted above, there is significant population structure in these inbred mice that is not present in either F2 or RI populations. This structure complicates the analysis, and in some cases prevents this strategy from being meaningfully applied to certain phenotypes. The association metric of our haplotype associationmethod also uses a relatively simple ANOVA model (in comparison to more complex maximum likelihood estimation). Traditional linkage analyses base their estimates on regression models that incorporate individual animals, whereas our ANOVA methodology utilizes strain means. Further, sizes of haplotype groups are small compared to the much larger number of individuals utilized in typical linkage studies. All of these factors can lead to a loss of power.

It is also important to note the strong dependence this haplotype association mapping method has on the inferred haplotype block structure in the mouse genome. While the existence of haplotype block structure is generally accepted, there is ongoing debate regarding the size of these blocks and the ability of haplotype association mapping methods to detect associations. More recent results of Frazer *et al*. [7] and Yalcin *et al*. [10] indicate the haplotype structure of inbred mice may contain regions of complexity which prevent even dense SNP maps from detecting meaningful associations between genotype and phenotype. We have also encountered this complexity when investigating certain loci that contain known quantitative trait genes. In some cases, we have observed that higher SNP densities are necessary to detect the known loci, possibly indicating a more fragmented haplotype in this region. Clearly those who utilize these algorithms must be cognizant of the limitations of their SNP set, but as SNP density increases the effect of these issues will be mitigated.

Regardless of the relative strengths and weaknesses between haplotype association and traditional QTL mapping, these methods are intermediate steps in pursuit of the final goal – identification of a gene which directly affects the phenotype of interest. In this study, we have used the HDLC levels as the primary phenotype to assess the performance of our algorithms. This phenotype was chosen because it has been extensively studied and many QTL have been previously identified. However, the list of QTL that influence HDLC levels are certainly not exhaustive, and in most cases the specific genes in the QTL regions have not been identified. While the ability of haplotype association methodologies to replicate loci identified in traditional QTL methods is encouraging, this comparison is not an ideal method to assess its specificity and sensitivity. Ultimately, a comprehensive assessment between these approaches may come only after the genetic basis for multiple complex traits has been exhaustively studied.

## Conclusion

The use of inbred strains of mice for QTL mapping has many advantages over traditional methods. However, there are also limitations in comparison to the traditional linkage analysis from F2 and RI lines, and application of these methods requires careful consideration of algorithmic choices based on both theoretical and practical factors. Here, we have demonstrated that the optimal choice of test-statistic depends on the structure of both the phenotypic and genotypic data, that the use of multi-SNP windows to infer local haplotype structure is essential when using this diverse population of inbred mouse strains, and that the gFWER approach is an effective way to control for multiple testing error while still preserving sensitivity.

## Authors' contributions

All authors participated in the design of the algorithms and interpretation of results. In addition, PMC performed all statistical analyses, and PMC and AS drafted the manuscript.

## Supplementary Material

Additional File 1 **Results of single-marker mapping (SMM) method for sweet taste preference. **The upper bar chart shows the computed HDLC phenotype association profile using the parametric SMM method. The lower bar chart shows the location of Tas1R3, a gene which has been previously shown to influence sweet taste preference [15]. The x-axis indicates the genomic axis, where chromosomal boundaries are indicated by the center bar. The maximum LOD scores are cut off at 12. Association scores below 3 and LOD scores below 3.3 are not shown. Peaks on the X chromosome are ignored, and multiple peaks within a 5 MB window are only counted once. Click here for file

Additional File 2 **Results of inferred-haplotype parametric (IH-P) method for sweet taste preference.** Click here for file

Additional File 3 **Results of inferred-haplotype Kruskal-Wallis (IH-KW) method for sweet taste preference.** Click here for file
