# Supplementary figures and images for: Comparative analysis of haplotype association mapping algorithms
Source: BMC Bioinformatics. 2006 Feb 9;7:61. doi: 10.1186/1471-2105-7-61 (PMC1409800; doi:10.1186/1471-2105-7-61)

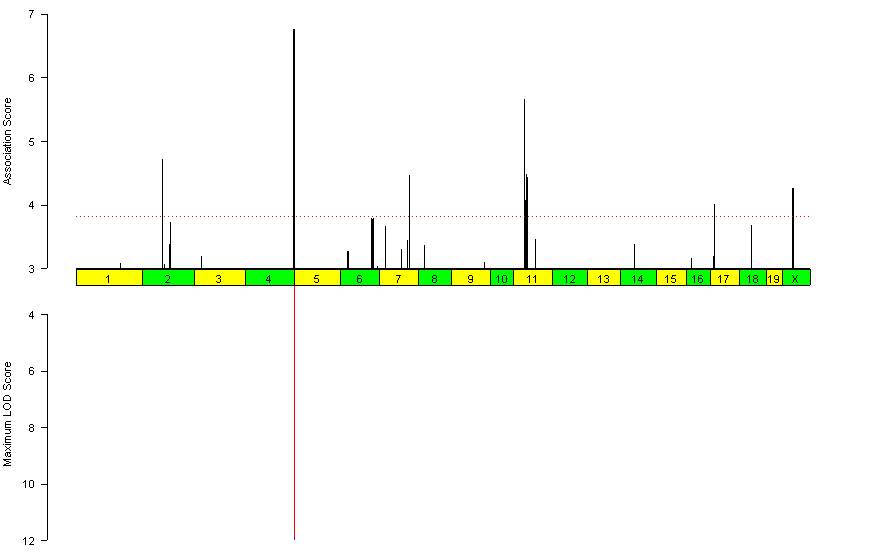

Supplement: Additional File 1 — Results of single-marker mapping (SMM) method for sweet taste preference. The upper bar chart shows the computed HDLC phenotype association profile using the parametric SMM method. The lower bar chart shows the location of Tas1R3, a gene which has been previously shown to influence sweet taste preference [15]. The x-axis indicates the genomic axis, where chromosomal boundaries are indicated by the center bar. The maximum LOD scores are cut off at 12. Association scores below 3 and LOD scores below 3.3 are not shown. Peaks on the X chromosome are ignored, and multiple peaks within a 5 MB window are only counted once. [file 1471-2105-7-61-S1.png]

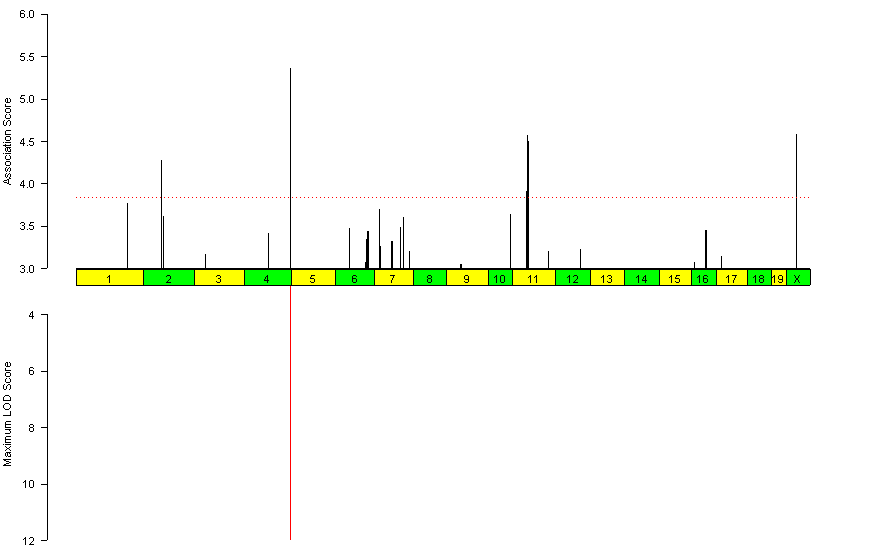

Supplement: Additional File 2 — Results of inferred-haplotype parametric (IH-P) method for sweet taste preference. [file 1471-2105-7-61-S2.png]

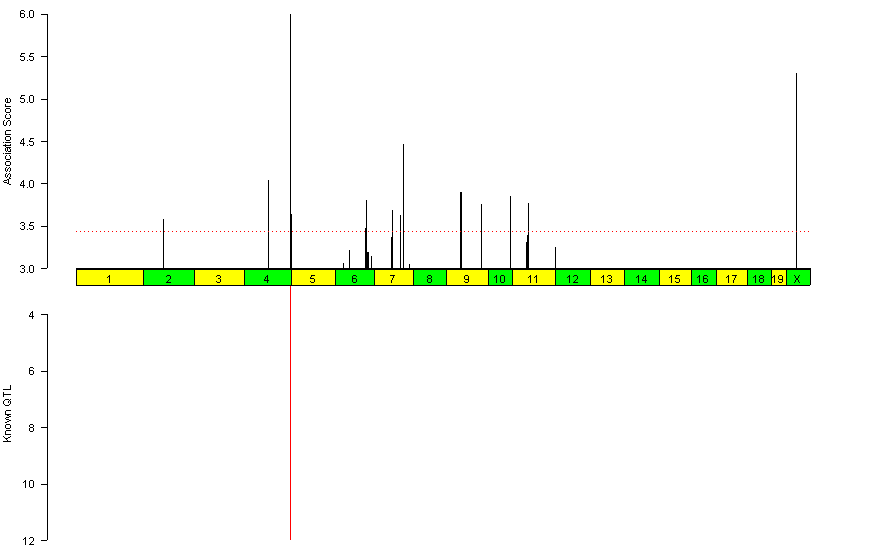

Supplement: Additional File 3 — Results of inferred-haplotype Kruskal-Wallis (IH-KW) method for sweet taste preference. [file 1471-2105-7-61-S3.png]
